# The impact of multimorbidity level and functional limitations on the accuracy of using self-reported survey data compared to administrative data to measure general practitioner and specialist visits in community-living adults

**DOI:** 10.1186/s12913-021-07160-2

**Published:** 2021-10-19

**Authors:** Lauren E. Griffith, Andrea Gruneir, Kathryn A. Fisher, Rumaisa Aljied, Richard Perez, Francis Nguyen, Christopher Patterson, Maureen Markle-Reid, Jenny Ploeg, Ross Upshur

**Affiliations:** 1 Department of Health Research Methods, Evidence, and Impact, McMaster University, MIP-Suite 309A, 1280 Main Street West Hamilton, Hamilton, Ontario L8S 4K1 Canada; 2 Department of Family Medicine, University of Alberta, Edmonton, Alberta Canada; 3 ICES, Toronto, Ontario Canada; 4 Women’s College Research Institute, Women’s College Hospital, Toronto, Ontario Canada; 5 School of Nursing, McMaster University, Hamilton, Ontario Canada; 6 ICES, McMaster University, Hamilton, Ontario Canada; 7 Department of Medicine, McMaster University, Hamilton, Ontario Canada; 8 Division of Clinical Public Health, Dalla Lana School of Public Health, University of Toronto, Toronto, Ontario Canada; 9 Bridgepoint Collaboratory for Research and Innovation, Sinai Health System, Toronto, Ontario Canada

**Keywords:** Healthcare utilization, Self-report, Administrative data, Agreement, Physician visits, Multimorbidity, Functional limitations

## Abstract

**Background:**

Researchers often use survey data to study the effect of health and social variables on physician use, but how self-reported physician use compares to administrative data, the gold standard, in particular within the context of multimorbidity and functional limitations remains unclear. We examine whether multimorbidity and functional limitations are related to agreement between self-reported and administrative data for physician use.

**Methods:**

Cross-sectional data from 52,854 Ontario participants of the Canadian Community Health Survey linked to administrative data were used to assess agreement on physician use. The number of general practitioner (GP) and specialist visits in the previous year was assessed using both data sources; multimorbidity and functional limitation were from self-report.

**Results:**

Fewer participants self-reported GP visits (84.8%) compared to administrative data (89.1%), but more self-reported specialist visits (69.2% vs. 64.9%). Sensitivity was higher for GP visits (≥90% for all multimorbidity levels) compared to specialist visits (approximately 75% for 0 to 90% for 4+ chronic conditions). Specificity started higher for GP than specialist visits but decreased more swiftly with multimorbidity level; in both cases, specificity levels fell below 50%. Functional limitations, age and sex did not impact the patterns of sensitivity and specificity seen across level of multimorbidity.

**Conclusions:**

Countries around the world collect health surveys to inform health policy and planning, but the extent to which these are linked with administrative, or similar, data are limited. Our study illustrates the potential for misclassification of physician use in self-report data and the need for sensitivity analyses or other corrections.

**Supplementary Information:**

The online version contains supplementary material available at 10.1186/s12913-021-07160-2.

## Background

Accurate healthcare utilization data are essential for making evidence-informed decisions for planning, prioritization, and health policy development. Data on physician visits, the most frequently used health service [1], commonly come from claims-based administrative and self-report sources [2]. Administrative data, particularly in jurisdictions with a single or large payer, are generally considered the reference standard for measuring health service use since they are typically complete and not vulnerable to concerns like recall bias. However, there are limitations in terms of access and population coverage. For example, in Canada most administrative data are available only at the province-level. In the United States, Medicare data are national but include only a subgroup of the population. Further, administrative data have limited information on the social determinants of health, physical function, symptoms, and other factors associated with healthcare utilization [3]. Large population-based health surveys, (such as the Canadian Longitudinal Study on Aging, Health and Retirement Study, and others collected in over 100 countries [4]) often include in-depth measures of these factors as well as self-report healthcare use data, which means they can be used to study the impact of a larger variety of variables on service use.

Multimorbidity has emerged as one of the greatest challenges facing healthcare [5, 6] and reliable estimates of its impact on physician use are key for planning. While authors have examined how well self-reported physician visits predict use based on administrative data, the results have been mixed [7]. This is not unexpected given the complexity of operationalizing multimorbidity and the potential for different kinds of conditions, such as symptomatic ones, to differentially impact on patient-important outcomes [8]. A systematic review demonstrated that people increasingly under-report their physician use relative to administrative data as their actual use increases [7] but research looking at the effect of multimorbidity, which is strongly associated with use, have found both under- and over-reporting [9–12]. No studies have examined the influence of multimorbidity on the accuracy of self-reported physician use, or if this might differ by the impact of those conditions on functional limitations. Globally, few of the over 100 population-based surveys collected worldwide are linked to administrative data [4, 13] meaning that efforts to study the effect of the socio-demographic, social, functional, or other variables on physician use most often rely solely on self-reported visits. However, if there is disagreement between data sources, and that disagreement is related to level of multimorbidity and/or functional limitations this could lead to biased estimates [14]. We undertook this study to: 1) Estimate agreement between self-reported physician visits and administrative data, 2) Examine whether agreement differs by level of multimorbidity and functional limitation, and 3) Examine whether any differences in agreement by level of multimorbidity and functional limitation may be explained by age and sex.

## Methods

### Study design and setting

This is a cross-sectional study in which we used population-based self-reported questionnaire and administrative data from Ontario, Canada’s most populous province [15]. Retrospective administrative data are used to match the timeframe (in the past 12 months) of the self-reported physician use questions. We followed the Strengthening the Reporting of Observational Studies in Epidemiology (STROBE) reporting guideline and the Reporting of Studies Conducted using Observational Routinely-Collected Health Data (RECORD) statement guidelines [16, 17].

### Data sources

General practitioner (GP) and specialist physician visits were obtained from provincial physician billing claims, which are generated for physician reimbursement, but also are used regularly for research and have been studied extensively for their validity [18].

Self-report data GP and specialist physician visits, chronic conditions, and functional limitations were obtained from the population-based Canadian Community Health Survey (CCHS). The CCHS collects information on health-related data at 2-year intervals from a random sample of community-living individuals 12 years or older. It covers approximately 97% of the target population in Canada and typically has response rates of > 70% [19]. Excluded from the CCHS sampling frame are persons living on First Nations reserves or Crown lands, in institutions, full-time members of the Canadian Forces, and residents of some remote regions. We pooled data from three CCHS cycles with consistent questions (2005–2006, 2007–2008, and 2009–2010) to increase the sample size.

For participants who consented (approximately 78% across the three cycles), CCHS data were linked to administrative data using unique encoded identifiers based on their CCHS participation date and analyzed at ICES. ICES is an independent non-profit research institute whose legal status under Ontario’s health information privacy laws allows it to collect and analyze health care and demographic data, without consent, for health system evaluation and improvement. The study received approval from the Hamilton Integrated Research Ethics Board at McMaster University (certificate # 13–590).

### Study participants

Of the 101,749 Ontario CCHS participants who agreed to linkage with administrative data, 54,893 were aged 45 years or older. We focussed on middle-aged and older adults as they have a higher prevalence of multimorbidity and are the focus of most multimorbidity research. We excluded participants receiving palliative care (*n* = 223) or residing in long-term care (124), had no healthcare contact in the previous 5 years (*n* = 68), were non-residents (*n* = 64), or ineligible for the Ontario Health Insurance Plan (*n* = 50). We further excluded participants with missing CCHS data on physician utilization, chronic conditions, or functional limitations (*n* = 1100), and non-matching age information between data sources (*n* = 136). If an individual was included in more than one CCHS cycle, we chose the first (*n* = 274). The final sample included 52,854 individuals.

### Measures of healthcare utilization

CCHS participants were asked if they had seen a family doctor or GP about their physical, emotional, or mental health in the past 12 months. If they responded “yes”, they were asked how many times. They were asked questions regarding specialists which were described as “any other medical doctor such as a surgeon, allergist, orthopaedist, or psychiatrist”. The 12-month timeframe is used in many population-based surveys in Canada, the United States (Health and Retirement Study and National Health Interview Survey) as well as the Survey of Health, Ageing and Retirement in Europe (SHARE) studies which were conducted in 28 European countries and Israel.

We compared the self-reported GP and specialist visits with those recorded in administrative data in the 12 months preceding the CCHS interview. To make the administrative data sources more comparable to the self-reported, we excluded billing for specialists who typically do not meet with patients (e.g., diagnostic radiologists). For both GP and specialist visits, we counted multiple billings within the same day as a single visit. We did not know if CCHS respondents reported their physician visit count as only those in outpatient clinic visits or if they included inpatient care. To assess this, we conducted sensitivity analyses in which we excluded specialist visits that occurred during hospital stays. We did not look at agreement on hospital stays, even though they are of high interest. CCHS participants were asked about overnight stays in hospital in the past 12 months, but the question does not allow us to distinguish between true hospital admissions and overnight stays in emergency departments or observation units.

### Multimorbidity and functional limitations

Multimorbidity was operationalized using 12 self-reported chronic conditions: Alzheimer’s diseases/dementia, anxiety/depression, arthritis, asthma, cancer, chronic obstructive pulmonary disease, diabetes, heart disease, hypertension, inflammatory bowel disease, stomach or intestinal ulcers, and stroke. These conditions were chosen because they are available in both data sources, prevalent in middle-aged and older adults [20], frequently reported in the multimorbidity literature [21], and consistent with our previous work [22–24]. In the CCHS, respondents were asked: “Has a doctor ever told you that you have [condition]?” and to consider conditions that lasted or were expected to last at least 6 months. Multimorbidity was defined as the sum of chronic conditions (0, 1, 2, 3, 4+). Having a functional limitation (yes or no) was defined as needing help with any of the following activities of daily living: preparing meals, appointments and errands, housework, personal care, moving inside house, and personal finances. While functional limitations data were only available in the CCHS, we chose to use multimorbidity based on self-report because most surveys are not linked to administrative data and we wanted to examine how in these cases measurable factors were associated with agreement.

### Covariates

Sex and age at CCHS interview date (categorized as: 45–54, 55–64, 65–74, and 75+) were identified using administrative data.

### Statistical analysis

We described demographics, multimorbidity, functional limitations, the average number of visits and the percent of participants reporting any GP and any specialist visit in the past 12 months based on self-report and administrative data both overall and by level of multimorbidity. For both GP and specialist visits, agreement between the two data sources on “any physician visit in the past 12 months” was measured by overall agreement (the percent whose self-report utilization in the past 12 months matched the administrative data), sensitivity (the percent with at least one physician visit in the past 12 months in administrative data who were correctly identified as having at least one physician visit based on self-report), and specificity (the percent of participants without a physician visit in the past 12 months who also self-reported no physician visits); administrative data was the reference. Sensitivity and specificity, with 95% confidence intervals, were calculated by level of multimorbidity and for those with and without functional limitations. We stratified by age and sex to examine for confounding and effect modification of observed patterns, given that both variables are associated with healthcare use [25], multimorbidity [26], and functional limitations [27]. A complete-case unweighted analysis was conducted using SAS 9.4 [28] as there was missing self-reported data for < 2% of eligible CCHS participants and CCHS weights are not available for the linkable subset of participants in Ontario .

## Results

We included 52,854 individuals aged 45 years or older who met our inclusion criteria (Supplemental Fig. 1). Of these, 29,593 (56.0%) were female and 22,839 (43.2%) were over age 65 (Table 1). Overall, 13,206 (25.0%) participants had no chronic conditions, 15,027 (28.4%) had one, and 24,621 (46.5%) had 2 or more; 8438 (16.0%) had functional limitations. The distribution of all characteristics was similar across the three CCHS cycles.

**Table 1:** Prevalence of demographic characteristics, number of chronic conditions, and functional limitations based on self-report for 52,854 Ontario Participants 45 years or older of the Canadian Community Health Survey Cycles 3–5

Characteristic	Cycle 3 2005/2006 (*n* = 16,382)	Cycle 4 2007/2008 (*n* = 18,424)	Cycle 5 2009/2010 (n = 18,048)	Total Cohort (*n* = 52,854)
Sex, n, (%) female	9184 (56.1)	10,212 (55.4)	10,197 (56.5)	29,593 (56.0)
Age Group, years, n (%)				
45–54	4548 (27.8)	4996 (27.1)	4437 (24.6)	13,981 (26.5)
55–64	4900 (29.9)	5619 (30.5)	5515 (30.6)	16,034 (30.3)
65–74	3794 (23.2)	4212 (22.9)	4282 (23.7)	12,288 (23.2)
75+	3140 (19.2)	3597 (19.5)	3814 (21.1)	10,551 (20.0)
Level of Multimorbidity, n (%)				
0	4124 (25.2)	4655 (25.3)	4427 (24.5)	13,206 (25.0)
1	4727 (28.9)	5253 (28.5)	5047 (28.0)	15,027 (28.4)
2	3734 (22.8)	4205 (22.8)	4064 (22.5)	12,003 (22.7)
3	2133 (13.0)	2463 (13.4)	2553 (14.1)	7149 (13.5)
4+	1664 (10.2)	1848 (10.0)	1957 (10.8)	5469 (10.3)
Functional Limitations, n (%)	2457 (15.0)	3013 (16.4)	2968 (16.4)	8438 (16.0)

Table 2 presents the data on GP and specialist visits in the past year based on self-report and administrative data. Compared to administrative data, participants under-reported both GP (mean 3.65 vs. 6.25) and specialist visits (mean 2.00 vs. 3.65) and the magnitude of the difference increased with the level of multimorbidity. Although the magnitude of the difference was smaller, a similar trend of increased under-reporting of specialist visits was still found when we excluded specialist visits that occurred during a hospital stay (data not shown). Compared to administrative data, participants under-reported having any GP visits (84.8% vs. 89.1%) but over-reported specialist visits (69.2% vs. 64.9%). Overall agreement, sensitivity and specificity were higher for GP visits compared to specialist visits. The percent visiting a GP and specialist increased with level of morbidity and the absolute difference between self-report and administrative data decreased. The overall agreement and sensitivity for both service types increased with level of multimorbidity but specificity decreased to a greater degree, especially for GP visits (0 vs. 4+ CCs: sensitivity 84.4% (83.7, 85.1%) vs. 95.7% (95.1–96.2%); specificity 71.7% (70.2, 73.3%) vs. 27.3% (19.3, 35.2%)).

**Table 2:** Number of GP and Specialist Visits Based on Self-Report and Administrative Data Sources by Number of Chronic Conditions

Health Service Use	0 CCs (***n*** = 12,206)	1 CC (***n*** = 15,027)	2 CCs (n = 12,003)	3 CCs (***n*** = 7149)	4+ CCs (***n*** = 5469)	Overall (n = 52,854)
**General Practitioner Visits**						
***Number of visits in past 12 months***						
Self-report						
Mean (SD)	1.77 (2.58)	3.00 (3.68)	4.12 (5.22)	5.28 (6.37)	6.84 (7.93)	36.5 (5.11)
Median (IQR)	1 (0, 2)	2 (1, 4)	3 (2, 5)	4 (2, 6)	4 (3, 10)	2 (1, 4)
Administrative data						
Mean (SD)	3.17 (3.97)	5.30 (5.63)	7.09 (6.72)	8.83 (7.63)	11.08 (9.30)	6.25 (6.79)
Median (IQR)	2 (1, 4)	4 (2, 7)	5 (3, 9)	7 (4, 11)	9 (5, 14)	5 (2, 8)
*Difference in number of visits (SR-Admin)*						
Mean (SD)	−1.39 (3.47)	−2.30 (5.15)	−2.97 (6.62)	−3.55 (7.70)	−4.24 (9.60)	−2.60 (6.24)
Median (IQR)	−1 (−2, 0)	− 1 (− 4, 0)	−2. (−5, 0)	−2 (−6, 0)	−3 (−7, 0)	−1 (− 4, 0)
***Any visit in past 12 months***						
Self-report, n (%)	9394 (71.1)	12,760 (84.9)	10,855 (90.4)	6630 (92.7)	5205 (95.2)	44,844 (84.8)
Administrative data, n (%)	10,081 (76.3)	13,473 (89.7)	11,311 (94.2)	6906 (96.6)	5348 (97.8)	47,119 (89.1)
*Raw Agreement*	81.4 (80.8, 82.1)	87.0 (86.4, 87.5)	89.9 (89.4, 90.4)	91.9 (91.3, 92.5)	94.2 (93.5, 94.8)	87.7 (87.4, 87.9)
*Sensitivity*	84.4 (83.7, 85.1)	90.1 (89.6, 90.6)	92.6 (92.1, 93.1)	93.8 (93.2, 94.4)	95.7 (95.1, 96.2)	90.7 (90.4, 90.9)
*Specificity*	71.7 (70.2, 73.3)	59.8 (57.4, 62.3)	45.4 (41.7, 49.1)	37.9 (31.8, 44.0)	27.3 (19.3, 35.2)	63.0 (61.7, 64.2)
**Specialist Visits**						
***Number of visits in past 12 months***						
Self-report						
Mean (SD)	1.10 (3.87)	1.67 (3.42)	2.24 (5.15)	2.74 (5.02)	3.61 (6.00)	2.00 (4.56)
Median (IQR)	1 (0, 1)	1 (0, 2)	1 (1, 3)	2 (1, 3)	2 (1, 4)	1 (0, 2)
Admin - Number of visits in past 12 months						
Mean (SD)	1.53 (3.49)	2.93 (5.46)	4.17 (6.70)	5.45 (7.85)	7.27 (9.69)	3.65 (6.55)
Median (IQR)	0 (0, 2)	1 (0, 4)	2 (0, 5)	3 (1, 7)	5 (2, 9)	1 (0, 5)
*Difference in number of visits (SR-Admin)*						
Mean (SD)	−0.43 (4.22)	−1.27 (4.64)	−1.94 (6.50)	−2.71 (7.48)	−3.67 (9.05)	−1.65 (6.10)
Median (IQR)	0 (−1, 1)	0 (−2, 0)	− 1 (−3, 0)	−1 (−4, 0)	−2 (−5, 0)	0 (− 2, 0)
***Any visit in past 12 months***						
Self-report, n (%)	7214 (54.6)	9945 (66.2)	9037 (75.3)	5741 (80.3)	4645 (84.9)	36,582 (69.2)
Administrative data, n (%)	5843 (44.2)	9307 (61.9)	8728 (72.7)	5737 (80.2)	4710 (86.1)	34,325 (64.9)
*Overall Agreement (95% CI)*	66.0 (65.2, 66.8)	69.4 (68.7, 70.2)	74.2 (73.4, 74.9)	78.7 (77.7, 79.6)	82.5 (81.5, 83.5)	72.3 (71.9, 72.6)
*Sensitivity (95% CI)*	73.3 (72.2, 74.5)	78.7 (77.9, 79.6)	84.0 (83.2, 84.8)	86.7 (85.9, 87.6)	89.2 (88.3, 90.1)	81.9 (81.5, 82.3)
*Specificity (95% CI)*	60.2 (59.1, 61.3)	54.2 (53.0, 55.5)	47.9 (46.2, 49.6)	45.8 (43.2, 48.4)	41.4 (37.9, 44.9)	54.3 (53.6, 55.1)

CC=Chronic Condition; CI=Confidence interval; In a 2 × 2 table representing any physician visit based on self-report (SR) and administrative (A) data in which cell a: yes (SR) and yes (A), cell b: Y (SR) and no (A), cell c: N(SR) and Y(A), and cell d: N(SR) and N(A), overall agreement = 100*((a + c)/(a + b + c + d)), sensitivity = 100*((a/(a + c)), and specificity = 100*((d/(b + d))

**Fig. 1:**
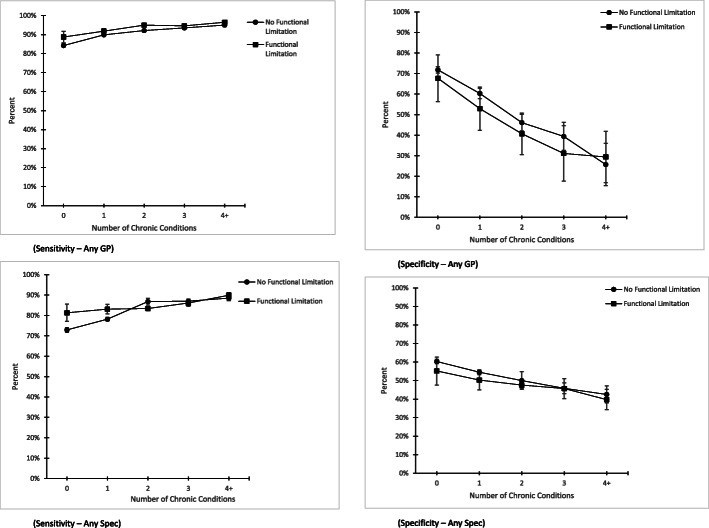
Sensitivity and Specificity of Any GP and Specialist Visit in the Previous 12 Months Based on Self-Report Compared to Administrative Data Stratified by Functional Limitation Status

In Fig. 1, we present the sensitivity and specificity of any GP and specialist visits based on self-report compared to administrative data by level of multimorbidity. The two lines represent participants with (squares) and without (circles) functional limitations. Regardless of the health service type, sensitivity increases and specificity decreases with level of multimorbidity with a similar pattern for people with and without functional limitations. There was some indication that people with functional limitations had slightly higher sensitivities for GP visits but the relationship was not consistent for specialist visits. There were no consistent differences in specificity for either GP or specialist visits.

**Fig. 2:**
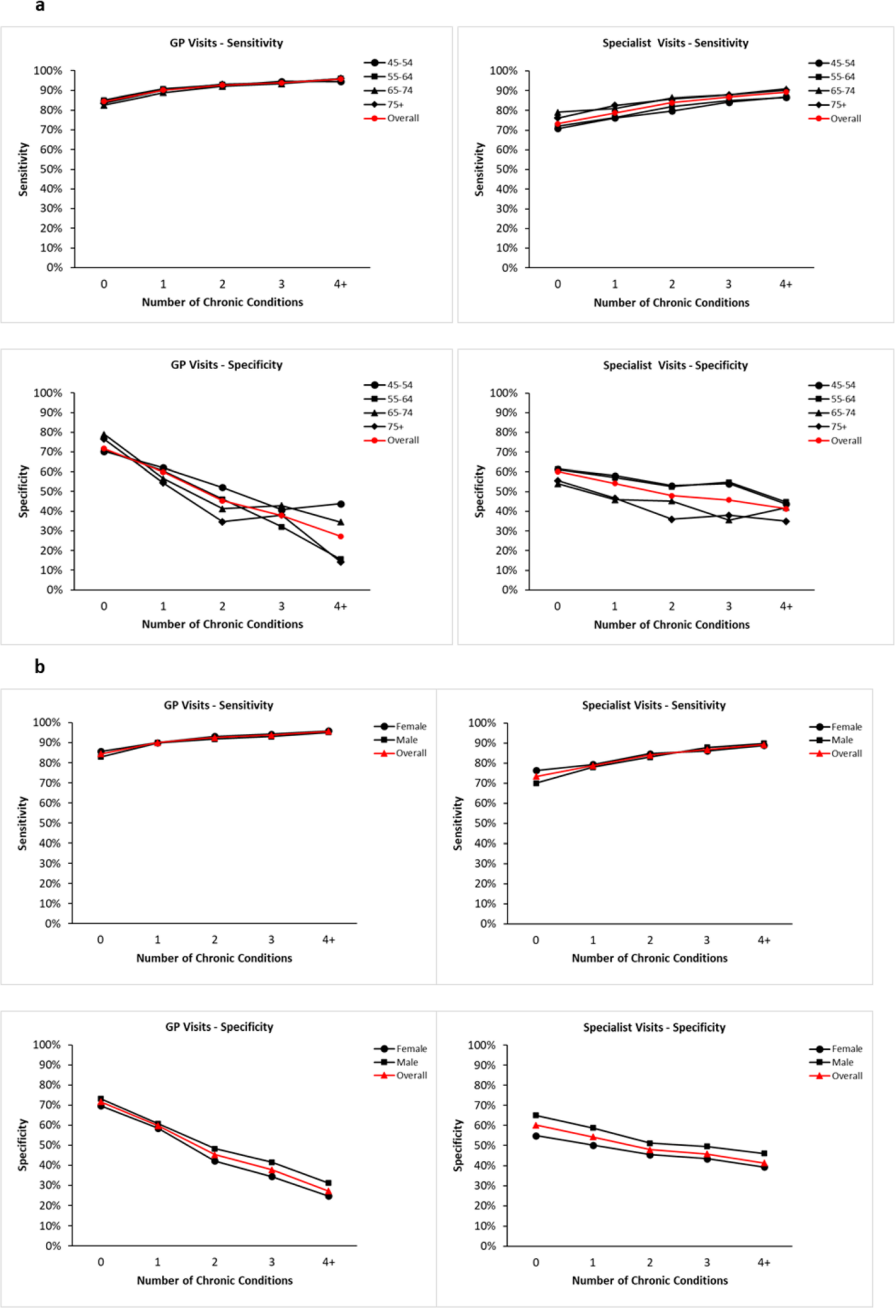
a b. Sensitivity and Specificity of Any GP and Specialist Visit in the Previous 12 Months Based on Self-Report Compared to Administrative Data Stratified by Age (2a) and Sex (2b)

We present sensitivity and specificity by level of morbidity stratified by age (Fig. 2a) and sex (Fig. 2b) with the overall line in red. Overall sensitivity was higher for GP visits (90% or higher for all levels of multimorbidity) compared to specialist visits (ranging from approximately 75% for 0 to 90% for 4+ chronic conditions). Specificity levels for self-report tended to start higher for GP visits than specialist visits but decreased more swiftly with multimorbidity level; in both cases specificity levels fell below 50%. There was some evidence that younger age groups and males had higher specificity for specialist visits, but the patterns across level of multimorbidity persisted indicating that the relationship is not likely due to confounding and that age and sex are not strong effect modifiers.

## Discussion

We sought to understand agreement between self-report and administrative data by level of multimorbidity. We found the average number of physician visits based on both data sources increased with level of multimorbidity, but self-report tended to underestimate physician use compared to administrative data. For both GP and specialist visits, the percent of participants with at least one encounter increased with the level of multimorbidity as did the sensitivity of self-reported data. In contrast, the specificity of self-reported data decreased with level of multimorbidity, especially for GP visits. The pattern in sensitivity and specificity did not differ greatly by functional limitations, but sensitivity was slightly higher for GP visits in those with functional limitations. Sex and age did not appear to be strong confounders or effect modifiers.

### Agreement on the number of physician visits

Prior studies [9, 11, 12, 29–31] generally found physician visits were under-reported compared to administrative data, but most combined GP and specialist visits. Of those that examined GPs and specialists separately, Raina et al. [29], who looked at individuals ≥ 65 years, found under-reporting for both GP and specialist visits while Peersman et al. [12], who looked at individuals ≥25 years, found under-reporting of GP visits but no difference in specialist visits. In a systematic review, Bhandari and Wagner [7] found under-reporting utilization was positively associated with increased utilization, thus under-reporting could be smaller in studies with younger adults, who tend to have fewer specialist visits. Our finding that under-reporting increased with multimorbidity, which is associated with age, supports this.

### Agreement on any physician visits

Our results for overall agreement and the relative under-reporting and over-reporting of “any” GP and “any” specialist visit mirrored Raina’s [29] and Peersman’s [12] results; however, Raina did not find a relationship between multimorbidity and discrepant reporting, which may have been because under- and over-reporting were treated as single “any disagreement” category due to sample size. While we found that sensitivity increased with the level of multimorbidity and specificity decreased to a greater extent, we did not find that these patterns differed by presence of functional limitations. Previous studies on agreement between healthcare utilization and functional limitations report mixed results [9, 11, 12]. We found some evidence for higher sensitivity (fewer false negatives) for self-reported GP visits in participants with functional limitations compared to those without, however this is likely no clinically meaningful, especially for those with two or more chronic conditions since sensitivity was greater than 90% in both groups.

Older age is one of the few sociodemographic factors that is consistently associated with under-reporting [7]. Sex, although not consistently found to impact on agreement, is associated with both the prevalence and nature of multimorbidity and the healthcare use experience [3]. We did not observe attenuation or consistent differences in the patterns of sensitivity and specificity across level of multimorbidity by age or sex suggesting that neither factor explains our findings.

### Limitations

We included 12 conditions in our definition of multimorbidity because we restricted to those available across all 3 CCHS cycles. Furthermore, our measure of functional limitations is fairly crude (any vs. none) and mainly focussed on instrumental rather than basic activities of daily living. Our finding of similar patterns in agreement between data sources for those with and without functional limitations could reflect that our measure did not adequately capture the phenomenon. Although we found differences in the relationship between chronic conditions and functional limitations using this operationalization in our previous work [32] future studies could examine more nuanced definitions of functional limitations when examining patterns of agreement. As well, we report only on the number of GP and specialist visits and not the need for these visits, whether health issues were addressed, or continuity of care. While much healthcare utilization research focuses on acute care, physician visits are the most common way in which people with multiple chronic conditions interact with the healthcare system. Finally, although there appears to be an association with increased levels of multimorbidity and agreement on healthcare utilization, we can not speculate as to the mechanism based on these data.

In conducting our study, a number of issues emerged that suggest that comparing administrative and self-report data is far more complex than would appear on the surface. For example, there is billing by physicians for services that patients do not see like diagnostic radiologists. In our analyses we also did not include hospitalizations because the language in the survey does not reflect how the information is captured in administrative data. This highlights the need for conceptual clarity when comparing across data sources to ensure that we are really asking the same thing from each. Finally, much of what we seek to understand is the burden of multiple chronic conditions – whether from the patient, provider, or system perspective – but frequently the full scope of care providers are not captured in administrative data (e.g. social workers or physiotherapists) while surveys rarely define these provider roles or capture the intensity of use. Neither data source on its own represents the totality of experience and, as is, can often be difficult to reconcile. Future surveys intended to link with or complement administrative data should consider how to ask questions that can be harmonized.

## Conclusions

Our agreement results may be generalizable to other population-based studies of community-living older adults as many have similar questions to assess chronic conditions and healthcare utilization, however patterns of utilization may vary among different healthcare settings. Both self-reported population-based data and administrative data are essential to understand healthcare utilization and its drivers, but globally few countries have access to population-based survey data linked with administrative data. While these data sources should complement one another, we found that they were not necessarily well-aligned. The results of our study can be used to better understand the potential impact of misclassification when using self-report data to measure physician visits. We found that among individuals with higher levels of multimorbidity who had no administrative record of seeing a physician in the prior year, a higher proportion self-reported having a seen a GP or specialist (false positive) than not (true negatives). This could attenuate estimates of association between multimorbidity and healthcare utilization based on self-report. Yet, large population-based surveys, like the CCHS, are vital for understanding drivers of service use because they capture a depth and breadth of data not available in administrative data. Although our data reflect the Canadian context, our findings illustrate the potential impact of misclassification in studies using self-reported physician utilization data and may guide strategies to address measurement error, as recommended by the STRengthening Analytical Thinking for Observational Studies (STRATOS) guidelines [33, 34] and used as a starting point for sensitivity analyses in other studies.

## Supplementary Information

**Additional file 1: Supplemental Fig. 1.** Study cohort creation including Ontario participants of the Canadian Community Health Survey cycles 3–5 who consented to administrative data linkage; CCHS, Canadian Community Health Survey, CC, chronic conditions

## Data Availability

ICES is a prescribed entity under the Ontario Personal Health Information Protection Act. As such, ICES policies and procedures are approved by Ontario’s Information and Privacy Commissioner. These policies require that access to data be limited to persons who require such access to perform their role on an approved ICES Project or Third-Party Project. Thus, we are prohibited from making ICES data publicly available. Only the results of analysis of ICES data may be made available. Researchers can contact das@ices.on.ca for data access.

## Abbreviations

CCHS: Canadian Community Health Survey
GP: General Practitioner
STRATOS: STRengthening Analytical Thinking for Observational Studies
