# Supplementary material for: The impact of multimorbidity level and functional limitations on the accuracy of using self-reported survey data compared to administrative data to measure general practitioner and specialist visits in community-living adults
Source: BMC Health Serv Res. 2021 Oct 19;21:1123. doi: 10.1186/s12913-021-07160-2 (PMC8527741; doi:10.1186/s12913-021-07160-2)
Supplement: Supplementary file 1 — Additional file 1: Supplemental Fig. 1. Study cohort creation including Ontario participants of the Canadian Community Health Survey cycles 3–5 who consented to administrative data linkage; CCHS, Canadian Community Health Survey, CC, chronic conditions [file 12913_2021_7160_MOESM1_ESM.docx]

**Supplemental Figure 1**

CCHS Participants (n=101,749)

Study Population

(n=52,854)

**Exclusions**

**Reason n**

<45 years of age 46,856

Missing data in CCHS 1,100

Multiple CCHS cycles 274

Palliative care recipients 223

Non-matching age in CCHS 136

Residents of Long-term care 124

No healthcare contact 68

Non-Ontario residents 64

Ineligible for OHIP 50

Supplemental Figure 1: Study cohort creation including Ontario participants of the Canadian Community Health Survey cycles 3-5 who consented to administrative data linkage; CCHS, Canadian Community Health Survey, CC, chronic conditions
